# The diagnostic value of quantitative parameters on dual-layer detector-based spectral CT in identifying ischaemic stroke

**DOI:** 10.3389/fneur.2023.1056941

**Published:** 2023-02-23

**Authors:** Jian Huang, Jinghua Chen, Ximing Wang, Ling Hao, Jinfeng Zhang, Xiaohui Zhang, Zhihong Sheng, Kefu Liu

**Affiliations:** ^1^Department of Radiology, Taicang Affiliated Hospital of Nanjing University of Traditional Chinese Medicine, Taicang, Jiangsu, China; ^2^Department of Radiology, Taicang Hospital, The Affiliated Hospital of Jiangsu Vocational College of Medicine, Yancheng, Jiangsu, China; ^3^Department of Radiology, First Affiliated Hospital of Suzhou University, Suzhou, Jiangsu, China; ^4^Department of Neurology, Taicang Affiliated Hospital of Nanjing University of Traditional Chinese Medicine, Taicang, Jiangsu, China; ^5^Clinical Science, Philips Healthcare, Shanghai, China; ^6^CT Marketing, Philips Healthcare, Nanjing, China; ^7^Department of Radiology, The Affiliated Suzhou Hospital of Nanjing Medical University, Suzhou, Jiangsu, China

**Keywords:** ischemic stroke, spectral CT, effective atomic number, iodine density values, iodine-no-water values

## Abstract

**Objective:**

To investigate the diagnostic value of quantitative parameters of spectral computed tomography (CT) in ischaemic stroke areas.

**Methods:**

The medical records of 57 patients with acute ischaemic stroke (AIS) who underwent plain computed tomography (CT) head scans, CT angiography (CTA), and CT perfusion (CTP) were retrospectively reviewed. The ischaemic areas (including the core infarct area and penumbra) and non-ischaemic areas in each patient were quantitatively analyzed using F-STROKE software. Two independent readers measured the corresponding values of the spectroscopic quantitative parameters (effective atomic number [Zeff value], iodine density value, and iodine–no-water value) in the ischaemic area and contralateral normal area alone. The differences in spectroscopic quantitative parameters between the two groups were compared, and their diagnostic efficacy was obtained.

**Results:**

The Zeff, iodine–no-water value, and iodine density value of the ischaemic area all showed significant lower than those of non-ischaemic tissue (*P* < 0.001). For differentiating the ischaemic area from non-ischaemic tissue, the area under the curve (AUC) of the Zeff value reached 0.869 (cut-off value: 7.385; sensitivity: 93.0%; specificity: 70.2%), the AUC of the iodine density value reached 0.932 (cut-off value: 0.235; sensitivity: 91.2%; specificity: 82.5%), and the AUC of the iodine–no-water value reached 0.922 (cut-off value: 0.205; sensitivity: 96.5%; specificity: 78.9%).

**Conclusion:**

The study showed the spectral CT would be a potential novel rapid method for identifying AIS. The spectral CT quantitative parameters (Zeff, iodine density values, and iodine–no-water values) can effectively differentiate the ischaemic area from non-ischaemic tissue in stroke patients.

Acute ischaemic stroke (AIS) is the second leading cause of death and the leading cause of disability worldwide. Ischaemia develops over time after the occurrence of AIS, so the acute management of AIS has important social and economic impacts (1, 2). From a historical point of view, PET imaging is known to be the gold standard for ischaemic stroke diagnosis (3), but PET is too time-consuming and inconvenient in a clinical setting. At present, studies (4) have found that diffusion-weighted imaging (DWI) is the best method to detect AIS under current clinical conditions. Currently, the early diagnosis of AIS relies on imaging studies, and commonly used methods include plain computed tomography (CT) scans of the head and CT vascular imaging (CT angiography; CTA) (5). CT perfusion (CTP) imaging may be undertaken when cerebral perfusion abnormalities are suspected, but not all patients will be examined with CTP. DWI is generally considered the most accurate imaging technique to assess the size of AIS in clinical settings (6). However, many AISs have a rapid onset and often go to the hospital at night. In many hospitals, night magnetic resonance (MR) examination is inconvenient or even impossible (7). In addition, MR examination has relatively stringent requirements for patients. Patients with cardiac pacemakers, pylorhophobia, or irritability cannot undergo this examination. Therefore, we need to find a method that can replace DWI examination to a certain extent.

In recent years, dual-layer, detector-based spectral CT has been introduced. SDCT employs a dual-layer detector in which lower energy photons are absorbed by the inner layer, and the higher energy photons are absorbed by the outer layer. Data from the two SDCT detector layers undergo spectral decomposition and are separated into photoelectric and Compton scatter components, which are used to generate spectral base images (SBI). SBI datasets can be used to reconstruct image sets emphasizing specific aspects of the spectral results. The use of a single source with simultaneous acquisition of lower and higher energy datasets in SDCT eliminates the potential for spatial and temporal misalignment. Spectral data are acquired for every scan, omitting the need for prospective protocol decisions regarding DECT mode. SDCT can obtain the same information as conventional CT scans while also providing dual-energy-based information with only one scan, without the need for a separate dual-energy scanning sequence. Compared with traditional CT, the advantages of SDCT mainly lie in its ability to obtain multiparameter imaging, single energy imaging, quantitative analysis, and energy spectrum analysis (8–10). This approach is easy to use and has gained clinical popularity. In addition, its multiple quantitative parameters have also been increasingly used in clinical settings (11–15). There are very few research reports on the diagnostic value of spectral parameters in AIS. This study provides a preliminary exploration of the diagnostic value of the quantitative parameters of spectral CT on ischaemic areas in AIS.

## Materials and methods

### Clinical data

Study materials were collected from the Suzhou Taicang Hospital of Traditional Chinese Medicine from November 2020 to August 2022. There were 178 cases where a diagnosis of AIS was made. The ethics committee of the Suzhou Taicang Hospital of Traditional Chinese Medicine approved this study and waived patient consent due to the retrospective study design (Grant No. 2022-023). All study procedures were carried out in accordance with the relevant guidelines and regulations.

**Figure 1:**
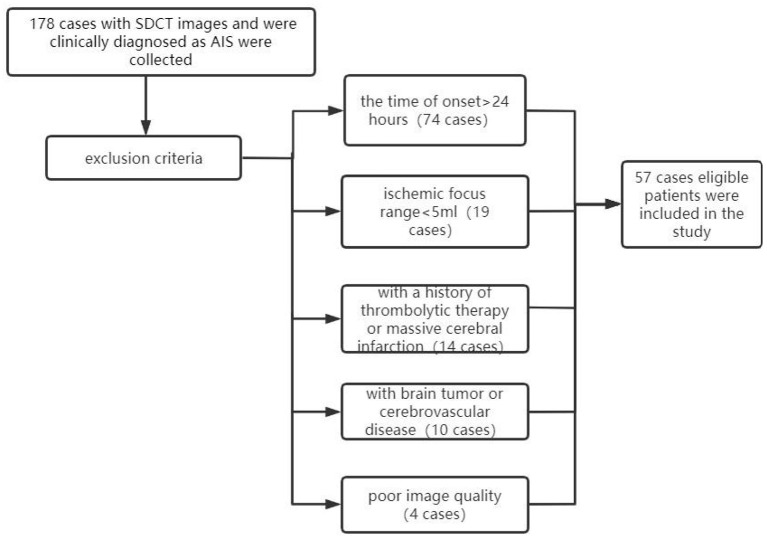
Flow chart of cases included/excluded in this study. SDCT, dual-layer spectral detector CT; AIS, acute ischaemic stroke.

Among these 178 cases, 57 patients who met the following criteria at our institution were retrospectively included in this study: (1) all patients clinically diagnosed with AIS had undergone one-stop, plain CT scan, CTP, and CTA, (2) all scans were performed within the 24 h of the onset of AIS, (3) all data had undergone valid quantitative analysis by F-STROKE (16), an automated perfusion analysis software (version 1.0.18; Neuroblast, Ltd. Co.), (4) all diagnostic images were clear, with no obvious motion artifacts or metallic artifacts evident, (5) no other brain diseases, such as brain tumors, vascular malformations, or cerebral hemorrhages, and (6) no previous history of thrombolytic therapy or massive cerebral infarction. Exclusion criteria included (1) the time of onset was >24 h, (2) the quantitative value was too small (ischaemic focus range: <5 mL), as determined using the F-STROKE software, (3) a history of thrombolytic therapy or massive cerebral infarction, (4) brain tumor or cerebrovascular disease, or (5) CT image quality did not meet the evaluation requirements (e.g., based on the presence of titanium clip artifacts or heavy motion artifacts). A flow chart of the inclusion/exclusion process is displayed in Figure 1. The CTA showed corresponding stenosis or occlusion in 50 cases and was normal in 7 cases.

### Demographic data

The studied population consisted of 57 AIS cases, as shown in the flow chart (Table 1). The median age was 66 years (IQR 50-88), and males accounted for 64.9% of patients. Thirty-six patients (63.1%) had hypertension, twenty-one patients (36.8%) had diabetes, twelve patients (21.1%) had coronary heart disease, ten patients (17.5%) smoked, five patients (8.7%) had previous stroke, and nine patients (15.8%) had alcoholism. The time of onset to door ranged from 30 to 1,380 min, with a median of 450 (IQR = 270–1,380) min. NIHSS at baseline ranged from 0 to 25, with a median of 7 (IQR = 3–10).

**Table 1:** Demographic data.

**Variables**	***N* = 57**
Age (median, IQR), years	66 (50–88)
Male (%)	64.9
Hypertension (%)	63.1
Diabetes (%)	36.8
Coronary heart disease (%)	21.1
Smoking (%)	17.5
Previous stroke (%)	8.7
Alcoholism (%)	15.8
Onset to door (median, IQR), min	450 (30–1,380)
Admission NIHSS (median, IQR)	7 (3–10)

IQR, inter quartile range.

### Spectral CT scanning methods

Scanning was performed using a Philips Healthcare IQon spectral CT machine (Best, the Netherlands). The patient lay in a supine position with the head advanced. The head was placed in the head frame of the examination bed, and the jaw was adducted so that the median sagittal plane of the head coincided with the midline of the bed and the auditory canthal line was perpendicular to the bed surface. Each patient was asked to keep their head immobile during the examination, avoiding swallowing movements and holding their head in position with a fixation plate before scanning. Axial scan mode was used for the plain CT scan and CTP, and energy spectral spiral scan mode was used for the CTA. The CT plain scan and CTA scan range remained consistent from the aortic arch to the cranial crest. The CTP scan range covered 8 cm. The CT plain scan was performed first, and then normal saline (20 mL) was injected through the right cubital vein at a flow rate of 5 mL/s using an Olympus high-pressure injector. After an additional injection of iodoform (50 mL; concentration: 350 mg/mL) at 5 s to initiate the CTP scan, and the interval between the end of the CTP scan was 5 min. This procedure was followed by an injection of saline (30 mL) and iodoform (45 mL; concentration: 350 mg/mL) at a 4.5 mL/s flow rate. Dynamic monitoring of the aortic arch region of interest (ROI) took place after the triggering threshold was reached (150 Hu). At this point, the CT machine automatically switched to spectral spiral scanning mode for the CTA examination, and the specific scanning parameters are shown in Table 2.

**Table 2:** CTP and CTA scan parameters.

**Parameters**	**CTA**	**CTP**
Layer thickness (mm)	5	5
Interlayer spacing (mm)	5	5
Tube voltage (km)	120	120
Tube current (mAs)	50	182
FOV (mm)	220	250
Collimator width (mm)	40	40
CTDIvol (mGy)	129.6	22.2
DLP (mGy*cm)	138.6	658

### Image analyses and evaluation

The ischaemic area (cerebral blood flow <30% of the normal value and Tmax >6 s) was automatically measured using F-STROKE perfusion software. As the obtained examination data were transferred to a dedicated workstation (spectral diagnostic suite [SPD]; Philips Healthcare), effective atomic number (Zeff) maps, iodine density maps, and iodine–no-water maps were obtained in real time in a post-processing workstation.

The Zeff characterizes the measured attenuation energy sensitivity of an unknown compound in terms of a resultant atomic number, which was estimated by the monochromatic attenuation ratio method since the monochromatic attenuation ratio is a monotonic as a function of effective atomic number (17, 18).

**Figure 2:**
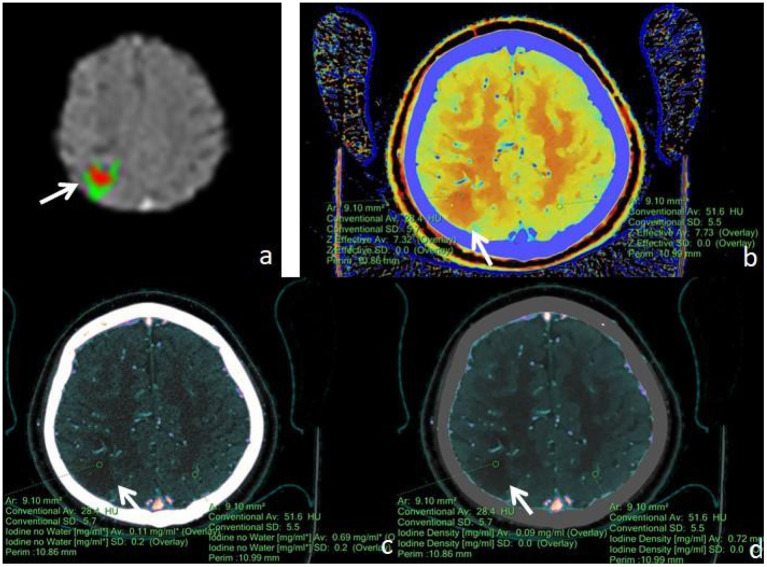
Male, 77 years old, was found with angular drooling and left-sided limb weakness for 6 h. **(a)** F-STROKE automated perfusion analysis software showed right parietal AIS with CBF <30% = 4.2 mL and T_max_ > 6S = 12.7 mL. **(b–d)** Zeff maps, iodine density maps, and iodine–no-water maps, respectively, with arrows indicating ischaemic areas in the right parietal lobe. **(b)** Measurement of an ischaemic area in the right parietal lobe with a Zeff value of approximately 7.32, and a corresponding Zeff value of approximately 7.73 in the contralateral non-ischaemic area. **(c)** Measurement of an absolute iodine value of approximately 0.11 mg/mL in the ischemic area of the right parietal lobe and a corresponding iodine–no-water value of approximately 0.69 mg/mL in the contralateral non-ischaemic area. **(d)** The iodine density value in the ischaemic area of the right parietal lobe was measured to be about 0.09 mg/mL, and the corresponding value in the contralateral, non-ischaemic area was measured to be about 0.72 mg/mL.

**Figure 3:**
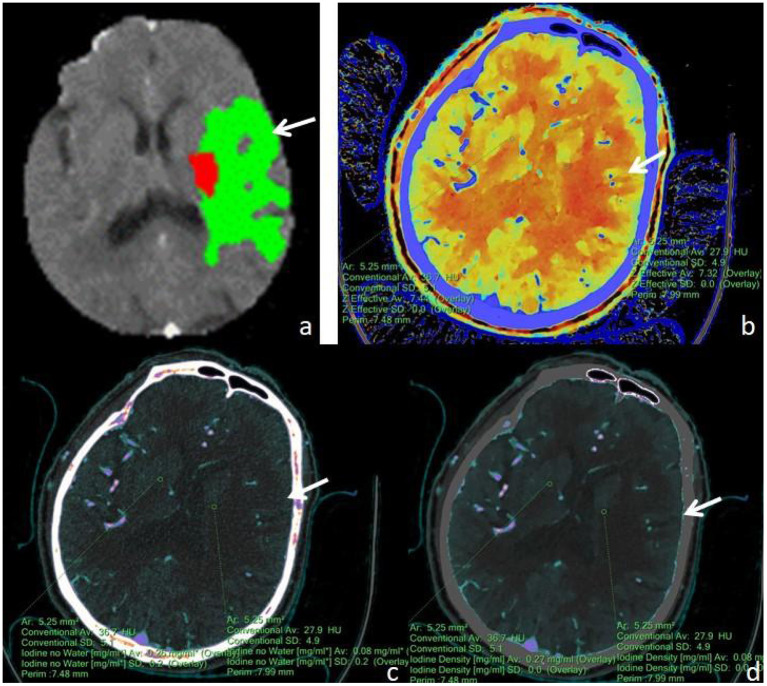
A male, 71 years old, presented with sudden aphasia with right-sided limb movement deficit for 8 h. **(a)** F-STROKE automated perfusion analysis software showed left temporal AIS with CBF <30% = 6.7 mL and Tmax >6S = 109.7 mL; **(b–d)** Zeff maps, iodine density maps, and absolute iodine maps, respectively, with arrows indicating ischaemic areas in the left temporal lobe. **(b)** A Zeff value of about 7.32 was measured in the ischaemic region in the left temporal lobe, while a value of about 7.44 was measured in the contralateral non-ischaemic region. **(c)** An iodine–no-water value of approximately 0.08 mg/mL was measured in the ischaemic area of the left temporal lobe, and a corresponding absolute iodine value of approximately 0.26 mg/mL was measured in the contralateral non-ischaemic area. **(d)** An iodine density value of approximately 0.08 mg/mL was measured in the ischaemic area of the left temporal lobe, and a corresponding iodine density value of approximately 0.27 mg/mL was measured in the contralateral non-ischaemic area.

**Figure 4:**
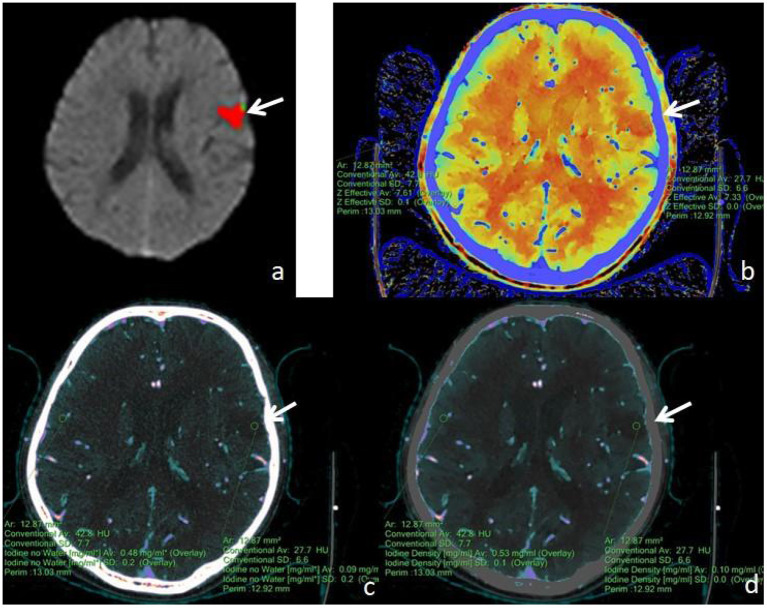
A male, 62 years old, presented with speech disorder and right-sided limb movement for 12 h. **(a)** F-STROKE automated perfusion analysis software showed left temporal lobe AIS with CBF <30% = 8.9 mL and Tmax >6S = 12.1 mL. **(b–d)** Zeff maps, iodine density maps, and iodine–no water maps, respectively, with arrows indicating ischaemic areas in the left temporal lobe. **(b)** A Zeff value of about 7.33 was measured in the ischaemic area in the left temporal lobe, and a corresponding Zeff value of about 7.61 was measured in the contralateral non-ischaemic area. **(c)** An absolute iodine value of approximately 0.09 mg/mL was measured in the ischaemic area of the left temporal lobe, and a corresponding absolute iodine value of approximately 0.48 mg/mL was measured in the contralateral non-ischaemic area. **(d)** An iodine density value of approximately 0.10 mg/mL was measured in the ischaemic area of the left temporal lobe, and a corresponding iodine density value of approximately 0.53 mg/mL was measured in the contralateral non-ischaemic area.

First, the brain perfusion defect area was identified according to the color difference on the fusion map of the iodine density map and effective atomic number. Then, referring to the results of F-STROKE software, the largest layer of the abnormal perfusion area was selected, and the ROI of the perfusion defect area and contralateral mirror brain area was manually drawn. We selected the core infarct area as the ROI and measured it manually three times to get the average value (Figures 2–4). The selection of ROI should avoid blood vessels and calcification as much as possible and then switch to the effective atomic number map, iodine density map and anhydrous iodine map. We measured and recorded the values of the spectroscopic quantitative parameters (effective atomic number [Zeff value], iodine identity value, and iodine-no water value) in the ischaemic area and comparative normal area. All measurements were performed independently by 2 radiologists who had more than 6 years of experience in neural imaging diagnosis. They were blinded to the spectral data measurements on the corresponding values of the spectroscopic quantitative parameters.

### Statistical methods

The statistical analysis was performed using SPSS version 26.0 (IBM Corporation, Armonk, NY, USA). The samples were tested for normality using the Shapiro–Wilk test. The samples were tested for homogeneity of variance using Levene's test, and continuous variables that were normally distributed and demonstrated homogeneity of variance were reported as the mean ± standard deviation ($\bar{x}$±s). Comparisons between groups were performed *via* two independent samples *t-test*s. Numerical variables that were not normally distributed were expressed as the median and interquartile range (IQR) and were compared between groups using the Mann–Whitney *U*-test. Receiver operating characteristic (ROC) curve analysis was used to predict efficacy, and cut-off values, sensitivity, and specificity were calculated. Intraclass correlation efficiency (ICC) was used to assess the level of interreader agreement.

## Results

The Zeff value of the ischaemic area was lower than that of the contralateral non-ischaemic area (7.35 ± 0.02 vs. 7.53 ± 0.01, respectively; *P* < 0.001). The iodine density value of the ischaemic area was lower than that of the contralateral non-ischaemic area (0.13 ± 0.02 vs. 0.42 ± 0.02, respectively; *P* < 0.001). The iodine–no-water value of the ischaemic area was lower than that of the contralateral non-ischaemic area (0.14 ± 0.02 vs. 0.45 ± 0.02, respectively; *P* < 0.001). The diagnostic efficacy of each spectral CT parameter for ischaemic lesions is shown in Table 3. Among all parameters, the highest value for iodine density was found for AIS (AUC = 0.932, cut-off = 0.235, sensitivity = 0.912, specificity = 0.825). ICC showed good agreement between two readers measuring Zeff, iodine density values, and iodine–no-water values for ischaemic areas and contralateral non-ischaemic areas (ICC 0.929, 95% CI 0.922–0.935; ICC 0.905, 95% CI 0.893–0.912; ICC 0.918, 95% CI 0.906–0.925).

**Table 3:** The diagnostic efficacy of the spectral parameters for ischaemic areas.

	**Zeff**	**Iodine density values (mg/mL)**	**Iodine–no-water values (mg/mL)**
AUC	0.869	0.932	0.922
*P*-value	0.000	0.000	0.000
Cutoff	7.385	0.235	0.205
Sensitivity	0.930	0.912	0.965
Specificity	0.702	0.825	0.789

## Discussion

AIS is one of the leading causes of morbidity and mortality worldwide, and multiple imaging modalities are used in the diagnosis and treatment of AIS, the most common of which are CT and MRI. With advances in imaging science and technology, the role of new imaging techniques in AIS evaluation continues to evolve (19, 20). At present, plain CT scan and CTA are still mainstays in the diagnosis of AIS (21), whereas CTP examination requires multiple repeat scans of the ROI, and patients receive significantly more radiation doses than conventional CT examination (22) and poor braking of some patients during dynamic continuous scanning may lead to failure of perfusion examination (23). Spectral CT (11–13, 24), as a new dual-energy CT imaging technique, provides additional information without the need for preselection, as per the CT protocol. It also avoids additional imaging, does not increase the radiation dose to the patient, allows for the use of lower amounts of contrast, enables spectral multiplication image reconstruction, and can be used for retrospective analysis. As such, its various applications in the clinic are convenient and fast.

Zeff maps are images specific to dual-energy CT. They are created with the underlying principle that the effective atomic number corresponding to the individual pixels is color quantified to form a pseudocolour map. The Zeff value differs from the CT value in that it adds information about the composition of matter for each pixel, reflecting substances with high atomic numbers, such as iodine, calcium, gadolinium, and substances with low atomic numbers, such as water and fat. These substances are easily resolved in effective atomic number maps (25). The present study found that the Zeff value derived by quantitative measurement had a sensitivity of 93.0% and a specificity of 70.2% for the discrimination of ischaemic and non-ischaemic zones. When the Zeff value was below 7.385, the possibility of considering ischaemic zones was large.

Spectral CT scans can show the perfusion status of normal and abnormal tissue regions by quantitative determination of iodine concentrations (26), where an iodine density map visually shows the distribution of iodine and allows for the quantitative determination of iodine concentration within the region of interest. Iodine density maps are optimal for lesions that absorb much iodine after contrast enhancement, and they are also advantageous for showing lesions that contain little iodine (27, 28). Water-free iodine maps (29) reflect areas where water-like tissues are identified and inhibited, enhancing the visualization of iodine-enhanced tissue. The pixel value in both images (in mg/mL) represents the iodine concentration of the displayed tissue, which can measure the iodine concentration of each pixel. In this study, we found that the difference between the ischaemic and non-ischaemic areas was significant, with cut-off values of 0.235 and 0.205, respectively, and that the AUC, sensitivity, and specificity were high, suggesting that the ischaemic area may be large when the value of iodine density is <0.235. The author believed that cerebral hypoperfusion existed in the early stages of the ischaemic area of AIS and that the filling of iodine contrast medium was reduced when compared with the normal, non-ischaemic area, and the corresponding iodine density and absolute iodine value decreased, consistent with the results of Fransson et al. (30) and other scholars.

In addition, in clinical work, most hospitals do not have automatic perfusion analysis software for CTP. This lack of software means that clinicians must perform manual programming, more complicated reconstruction, and post-processing. Image interpretation is thus complicated and time-consuming, whereas the plain CT + CTA imaging protocol has provided sufficient information about strokes to aid in clinical decision-making, save time, and reduce radiation doses. The results of the present study indicate that during a single CT examination, the irradiated dose (a dose length product [DLP] of approximately 658 mGy ^*^ cm) in CTA is much lower than that of CTP at an irradiated dose of 1,038.6 mGy ^*^ cm. The radiation dose needed for CTA is lower than that required for CTP, and it is not susceptible to factors such as the posttreatment platform used and the head motion of patients, among others (31–34). If CTA can provide the same information as CTP, it is unnecessary to perform both examinations at the same time. Since CTP usually does not use the same amount of contrast agent as CTA^31^, the scanning process will allow the radiation dose and contrast agent dosage to be reduced simultaneously. Yuto Uchida (35) used magnetic resonance oxygen extraction fractions to predict ischaemic penumbra volume. Therefore, whether spectral CT quantitative parameters can effectively predict the ischaemic penumbra and core infarct area needs to be explored and further studied.

There are limitations in this study. First, the number of patients included in this study was relatively small, and the patients were from only one clinical center. Second, this study is retrospective and may have selection bias. Finally, this study did not use thresholds for external validation of the prediction results, and additional research needs to be conducted across different research institutions to validate the results of this study.

## Conclusion

The study showed the spectral CT would be a potential novel rapid method for identifying AIS. The spectral CT quantitative parameters (Zeff, iodine density values, and iodine–no-water values) can effectively differentiate the ischaemic area from non-ischaemic tissue in stroke patients.

## Data availability statement

The raw data supporting the conclusions of this article will be made available by the authors, without undue reservation.

## Ethics statement

The studies involving human participants were reviewed and approved by the Ethics Committee of the Suzhou Taicang Hospital of Traditional Chinese Medicine. The Ethics Committee waived the requirement of written informed consent for participation.

## Author contributions

KL contributed to the conception of the study and writing-review and funding acquisition. JH contributed significantly to original draft and supervision. JC contributed equally to original draft and supervision. XW contributed significantly to perform the analysis with constructive discussions. JZ contributed significantly to the data curation. XZ contributed significantly to manuscript preparation. ZS contributed significantly to software. All authors contributed to the article and approved the submitted version.
